# The ATPase Pontin is a key cell cycle regulator by amplifying E2F1 transcription response in glioma

**DOI:** 10.1038/s41419-021-03421-4

**Published:** 2021-02-01

**Authors:** Run Wang, Xuebing Li, Cuiyun Sun, Lin Yu, Dan Hua, Cuijuan Shi, Qian Wang, Chun Rao, Wenjun Luo, Zhendong Jiang, Xuexia Zhou, Shizhu Yu

**Affiliations:** 1 Department of Neuropathology, Tianjin Neurological Institute, Tianjin Medical University General Hospital, Tianjin, China; 2 Tianjin Key Laboratory of Injuries, Variations and Regeneration of the Nervous System, Tianjin, China; 3 Key Laboratory of Post-trauma Neuro-repair and Regeneration in Central Nervous System, Ministry of Education, Tianjin, China; 4 Tianjin Key Laboratory of Lung Cancer Metastasis and Tumor Microenvironment, Tianjin Lung Cancer Institute, Tianjin Medical University General Hospital, Tianjin, China; 5 Department of Biochemistry and Molecular Biology, School of Basic Medical Sciences of Tianjin Medical University, Tianjin, China

**Keywords:** Cell growth, CNS cancer

## Abstract

Pontin (RUVBL1) is a highly conserved ATPase of the AAA + (ATPases Associated with various cellular Activities) superfamily and is implicated in various biological processes crucial for oncogenesis. Its overexpression is observed in multiple human cancers, whereas the relevance of Pontin to gliomagenesis remains obscure. To gain insights into Pontin involvement in glioma, we performed bioinformatics analyses of *Pontin* co-expressed genes, Pontin-affected genes, and carried out experimental studies. The results verified that Pontin was upregulated in gliomas. Its higher levels might predict the worse prognosis of glioma patients. The *Pontin* co-expressed genes were functionally enriched in cell cycle progression and RNA processing. In the nucleus, Pontin promoted cell growth via facilitating cell cycle progression. Using RNA-seq, we found that Pontin knockdown resulted in altered expression of multiple genes, among which the E2F1 targets accounted for a large proportion. Mechanistic studies found that Pontin interacted with E2F1 and markedly amplified the E2F1 transcription response in an ATPase domain-dependent manner. By analyzing the RNA-seq data, we also found that Pontin could impact on the alternative splicing (AS). Both differential expressed genes and AS events affected by Pontin were associated with cell cycle regulation. Taken together, our findings provide novel insights of the importance of Pontin in gliomagenesis by regulating cell cycle and AS, and shed light on the possible application of Pontin as an antineoplastic target in glioma.

## Introduction

Gliomas are the most common primary brain tumors^1^. Malignant glioma, especially glioblastoma (GBM, WHO grade IV glioma), is characterized by excessive proliferation, diffuse infiltration into the surrounding brain tissue, and antitumor immune escape^2,3^. Surgical resection followed by traditional radiotherapy plus chemotherapy is the standard treatment strategy for malignant gliomas^4,5^. Unfortunately, gliomas often relapse in almost all the patients, and the prognosis of GBM patients remains dismal^2,6^. Therefore, the underlying molecular mechanisms involved in gliomagenesis need to be explored more in detail to improve the treatment effects of this devastating disease.

Pontin, officially named as RUVBL1, belongs to the highly conserved AAA + (ATPase Associated with diverse cellular Activities) ATPase superfamily. This protein family is characterized by having the conserved Walker A and Walker B motifs in the ATPase domain, which are responsible for ATP binding and hydrolysis^7^. Pontin is a component of several protein complexes and is involved in multiple cellular processes, including cell cycle/mitotic progression, transcription regulation, chromatin remodeling, DNA damage response, assembly of macromolecular complexes, and cellular motility, all of which contribute to its central roles in promoting cell growth^8^. Notably, Pontin was initially identified as an interactor of various nuclear factors, including TIP60, *β*-catenin, and c-Myc^9–11^, and it is a crucial oncoprotein in cancers^12^. For instance, as a subunit of the TIP60 complex, Pontin is required for the histone acetyltransferase activity of TIP60, and is highly implicated in the regulation of this activity^13^. Pontin regulates β-catenin-mediated TCF-target gene expression via modulating the chromatin structure and the transcription activity^14^. The interaction of Pontin with c-Myc provides a linkage of Pontin with c-Myc-dependent biological activities, including oncogenic transformation, apoptosis inhibition, and differentiation blockage^15^. Furthermore, Pontin is frequently overexpressed in many human cancers, and acquires activating mutations upstream or downstream of its functional pathways, suggesting the important roles of Pontin in tumorigenesis and cancer development^16^. However, the possible roles of Pontin in glioma remain currently unexplored.

Previous studies in a variety of human cancers have consistently concluded that inhibition of Pontin could result in cell growth retardation and cell cycle arrest at the G1/S checkpoint^8^. G1/S transition is accurately controlled by a series of proteins/pathways that maintain resting until S phase entry is signaled^17^. For instance, the transcription factor E2F1, which switches on the expression of various S-phase promoting genes, is kept in the inactivation status by retinoblastoma proteins (RB)^18^. Phosphorylation by cyclin D1 makes E2F1 dissociation from RB, therefore activates the transcription of the E2F1 targets^19^. Notably, Pontin may function as a co-activator of E2F1 by directly interacting with E2F1, opening the chromatin structures at the E2F1 target genes, and amplifying the transcriptional response of cell cycle and metabolic genes during cancer progression^20,21^. However, it remains unknown whether this cooperation exists in glioma.

Alternative splicing (AS) generates different mRNA isoforms from a single primary transcript. Over 95% of human genes undergo AS to contribute to the protein diversity^22^. Genome-wide studies indicate that dysregulated AS is highly implicated in the carcinogenesis of various types of human cancers^23,24^. Previous reports found that Pontin could associate with spliceosomal U4 and U5 small nuclear ribonucleoproteins (snRNPs), and was involved in snRNP assembly or trafficking^25^. The knockdown of Reptin (officially named as RUVBL2), a close family member of Pontin, significantly affected the AS profiles^26^. These clues indicate a possible implication of Pontin in AS regulation.

In the present study, by using online resources, performing cellular experiments and RNA-seq, we investigated the expression, biological functions, and molecular mechanisms of Pontin in gliomagenesis. Our results revealed that Pontin was overexpressed in gliomas and its higher levels predicted the worse prognosis of glioma patients. The *Pontin* co-expressed gene network analysis indicated a critical role of Pontin in cell cycle control and RNA processing in glioma. Moreover, knockdown of Pontin in GBM cells resulted in cell cycle arrest and growth retardation. A further examination verified that Pontin majorly amplified the E2F1 transcription response as a co-activator of E2F1, in which the ATPase domain of Pontin was indispensable. Strikingly, the RNA-seq results indicated the relevancy of Pontin with AS regulation. These findings provide novel insights into the oncogenic roles of Pontin in glioma, and raise the possibility of Pontin as a valuable prognostic factor and potential target in glioma therapy.

## Materials and methods

### Specimens and cell culture

Resected specimens of 30 astrocytic gliomas (grade II, III, IV) and 10 non-tumoral brain tissues (control) were collected from Tianjin Medical University General Hospital (TMUGH). Specimens are pathologically graded according to the 2016 WHO criteria^3^. These specimens were immediately fixed in 3.7% buffered formaldehyde solution after surgical excision and embedded in paraffin. Five micrometers continuous sections were then prepared for immunohistochemistry (IHC) of Pontin. Another cohort of resected specimens including 30 GBMs and 5 control tissues were also obtained from TMUGH, and preserved in liquid nitrogen and stored at −80 °C until used for RNA isolation. This study has been approved by the Ethics Committee of TMUGH, and all participants signed informed consent. Human GBM cell line U87MG was obtained from the American Type Culture Collection (ATCC; USA). LN229 and U251 cell lines were purchased from the China Academia Sinica Cell Repository (Shanghai, China). All the cell lines were grown in the complete Dulbecco’s Modified Eagle Medium (DMEM; Gibco, USA) supplemented with 10% fetal bovine serum (FBS; Gibco) at 37 °C with 5% CO_2_ atmosphere in a humidified incubator.

### In silico analysis of *Pontin* expression and *Pontin* co-expressed genes in glioma

We analyzed the pan-cancer expression profile of *Pontin* using the Gene Expression Profiling Interactive Analysis (GEPIA) dataset (http://gepia.cancer-pku.cn). Pontin expression in gliomas was also analyzed by the Oncomine dataset (https://www.oncomine.org). cBioPortal (http://www.cbioportal.org) was used to identify the *Pontin* co-expressed genes in GBM. Briefly, the threshold selected was the absolute value of Spearman’s correlation coefficient >0.5. The online tool STRING (http://string-db.org) was applied to identify the potential interaction network and functional enrichments (gene ontology (GO) annotation, KEGG pathways) of protein products of these co-expressed genes.

### SiRNAs, lentiviruses, and stable sub-cell line establishment

The two independent siRNAs targeting Pontin (si-Pontin-1: 5′-CCAUGCUGUAUACUCCAC AGGAAAUdTdT-3′; si-Pontin-2: 5′-UAAAGGAGACCAAGGAAGUdTdT-3′) and the negative control (si-nc: 5′-GGUGGAACAAUUGCUUUUAdTdT-3′) oligonucleotides were synthesized by Gene Pharma (Suzhou, China), and used to transfect U87MG, LN229, and U251 cells with HiperFect^®^ Transfection Reagent (QIAGEN, Germany) as per the standard protocol. Recombinant lentiviruses expressing sh-Pontin and negative control (sh-NC) were purchased from GeneChem (Shanghai, China). U87MG stable sub-cell lines were established by infecting with the indicated lentivirus and selecting with puromycin.

### Western blot and Co-immunoprecipitation (Co-IP)

Western blot was carried out as previously described^27^. The primary antibodies used in Western blot were as follows: mouse anti-human Pontin (catalog SAB4200194; Sigma-aldrich, USA), mouse anti-human GAPDH (catalog BM3876; Boster, Wuhan, China), rabbit anti-human E2F1 (catalog 49286; Sabbiotech, USA), rabbit anti-human CDK1 (catalog 10762-1-AP; Proteintech, USA), rabbit anti-human CDK4 (catalog 12790 T; Cell Signaling Technology, USA), mouse anti-human cyclin B2 (catalog sc-28303; Santa Cruz Biotechnology, USA), mouse anti-flag tag (catalog F3165; Sigma-aldrich, USA). Co-IP was performed as previously described^28^. In brief, cell extracts were immunoprecipitated with each 2 μg of indicated antibody (IgG, E2F1, or flag antibody) for 4–6 h and with protein A/G agarose beads (catalog No. sc-2003; Santa Cruz Biotechnology) overnight. Bound proteins were then washed, resuspended in protein sample buffer, separated by SDS-PAGE and detected by immunoblot.

### RNA isolation and quantitative RT-PCR (qRT-PCR)

Total RNA was isolated from tissues or cells utilizing TRIzol reagent (Invitrogen, USA) according to the manufacturer’s instructions. After reverse transcription, qRT-PCR was performed using GoTaq^®^qPCR Master Mix (Promega, USA). The primers are listed in Supplementary Table S1. ACTB was used as the housekeeping gene. The relative mRNA expression levels (fold change) were calculated by the 2^–ΔΔCt^ method and each experiment was performed in triplicate.

### Cell growth assay

Cell growth was determined by 3-(4, 5-dimethylthiazol-2-yl)-5- (3-carboxymethoxyphenyl)-2-(4-sulfophenyl)-2H-tetrazolium (MTS) assay as described previously^29^. Briefly, 2 × 10^3^ cells were seeded in 96-well plates per well. Upon analysis, 100 µL completed medium containing 20 µL of MTS (Cell Titer 96^®^AQeuous One Solution Reagent; Promega) was added into each well, and the cells were incubated for another 2 h at 37 °C. The absorbance of each well was measured with a microplate reader at the wavelength of 490 nm. The surviving cells were measured every day for four consecutive days.

### Cell cycle analysis

Cell cycle was analyzed by propidium iodide (PI) staining as described previously^29^. In brief, cells were digested, washed with PBS, and fixed in the ice-cold 70% ethanol at −20 °C overnight. Then cells were stained with 40 μg/mL of PI staining buffer (Sigma) at room temperature for 30 min. Following analysis by the Accuri C6/FACSCalibur Flow Cytometer (BD Biosciences, USA), cell cycle data were processed with ModFit LT software (Verity Software House). All experiments were performed in triplicate.

### Immunofluorescence staining

Immunofluorescence (IF) staining was performed following the standard procedures as described previously^28^. The antibodies used in IF were as follows: mouse anti-human Pontin antibody (catalog SAB4200194; Sigma-aldrich), rabbit anti-human E2F1 antibody (catalog 49286; Sabbiotech), FITC-labeled goat anti-mouse IgG secondary antibody (catalog A-16079; Thermo Fisher Scientific, USA), TRITC-labeled goat anti-rabbit IgG secondary antibody (catalog A-16101; Thermo Fisher Scientific). DAPI reagent was used to visualize the cell nuclei.

### Plasmids, transfection, and luciferase reporter assay

To confirm the Pontin domain interaction with E2F1, the shRNA-resistant full-length (fl) human Pontin and its domain deletion mutants were constructed using pCMV-tag2b vector (flag tagged). To perform the luciferase reporter assay, a plasmid expressing wild-type Pontin or E2F1 was constructed using pcDNA3.1. The AURKA –1700 and −255 luciferase reporter plasmids were constructed by cloning the respective promoter region (−1700 to −200 for AURKA −1700; −255 to −200 for AURKA −255) of AURKA gene into the pGL3-basic vector (Promega). E2F1-binding sites within the AURKA promoter region were identified by the Jaspar database (http://jaspar.binf.ku.dk/cgi-bin/jaspar_db.pl). All the plasmid transfection was done with Lipofectamine 3000 (Invitrogen, USA). The cloning primers were listed in Supplementary Table S2. Luciferase reporter assay was done as per the standard procedures as described previously^29^. Values are expressed as means ± SD of three independent experiments.

### RNA-seq and data analysis

Total RNA isolated from control (sh-NC) and Pontin knockdown (sh-Pontin) groups of U87MG cells were subjected to paired-end RNA-Seq using Illumina HiSeq 2000 system. Reads mapping and data analysis for differential expressed genes, differential AS events were carried out as previously described^30^.

### Statistical analysis

The statistical tests for data analysis included 2-tailed Student’s *t* test, one-way ANOVA test, Pearson correlation analysis, Kaplan–Meier analysis, and log-rank test. Statistical analyses were performed using SPSS 21.0 software (IBM, Carlsbad, CA, USA). The data were presented as the mean ± SD; *P* ⩽ 0.05 was considered statistically significant. All the experiments of cell lines were performed at least three times with triplicate samples.

## Results

### Pontin was overexpressed in gliomas and its higher expression predicted worse prognosis

**Fig. 1:**
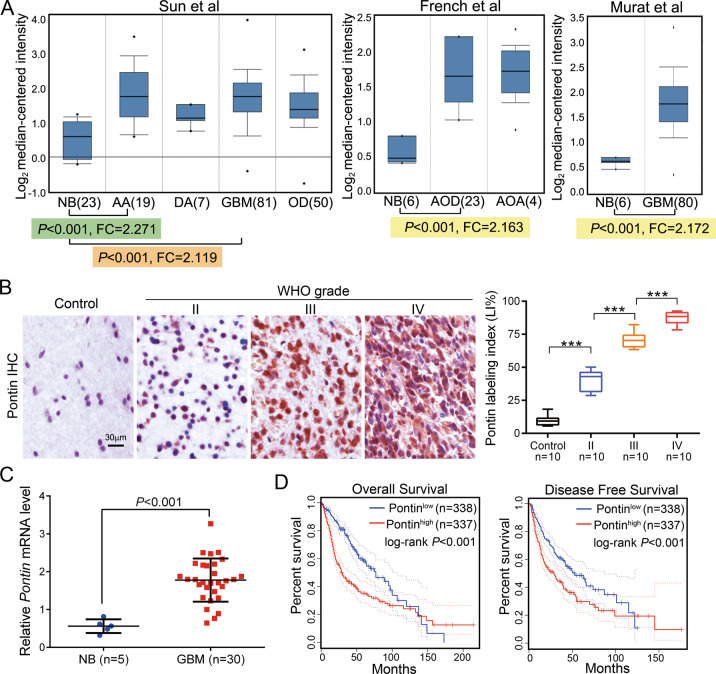
Increased expression of Pontin in gliomas. **A** Box plots comparing *Pontin* mRNA levels between normal brains (NB) and gliomas using the data from Oncomine. Boxes represent the 25th and 75th percentiles, lines represent the median, and whiskers show the minimum and maximum points. Sun et al: NB (*n* = 23), anaplastic astrocytoma (AA, *n* = 19, grade III), diffuse astrocytoma (DA, *n* = 7, grade II), GBM (*n* = 81, grade IV), oligodendroglioma (OD, *n* = 50, grade II). French et al: NB (*n* = 6), anaplastic oligodendroglioma (AOD, *n* = 23, grade III), anaplastic oligoastrocytoma (AOA, *n* = 4, grade III). Murat et al: NB (*n* = 6), GBM (*n* = 80, grade IV). Data are expressed as normalized expression units. FC, fold change. **B** Representative IHC staining for Pontin in graded gliomas (left) and quantification of Pontin expression levels [Labeling index (%)] among groups of the FFPE samples (right). Scale bar, 30 μm. Data are presented as the mean ± SD. **C** qRT-PCR detection of *Pontin* mRNA expression in human NBs (*n* = 5) and GBMs (*n* = 30) collected from our hospital. Data are presented as the mean ± SD. **D** Kaplan–Meier analyses of the overall survival (left) and disease-free survival (right) of all the glioma patients from the GEPIA dataset. Patients were stratified into high and low expression groups using the median of Pontin expression as cutoffs.

To investigate the implication of Pontin in cancer development, we firstly analyzed its expression by utilizing the public data from the GEPIA database. The expression profile across various cancer types and the matched normal tissues indicated that Pontin was significantly increased in a majority of cancers, including in brain GBMs and lower grade gliomas (LGGs; Supplementary Fig. S1). To further evaluate the expression of Pontin in gliomas, we also analyzed its expression using the Oncomine database. As shown in Fig. 1A, *Pontin* mRNA levels were markedly higher in different subtypes of gliomas than in normal brains (NBs). Consistently, IHC showed the increased Pontin expression in gliomas, with its labeling indexes progressively increasing along with the glioma grades elevation (Fig. 1B). Subsequent qRT-PCR results also indicated that *Pontin* was significantly upregulated in GBMs than that in the NBs (Fig. 1C). In addition, Kaplan–Meier analysis revealed that glioma patients with higher Pontin expression clearly had worse overall survival (OS) and disease-free survival (DFS; Fig. 1D). These data demonstrated that Pontin was significantly overexpressed in gliomas, and its overexpression might predict worse prognosis of the glioma patients.

### *Pontin* co-expressed genes were functionally associated with cell cycle control and RNA processing

**Fig. 2:**
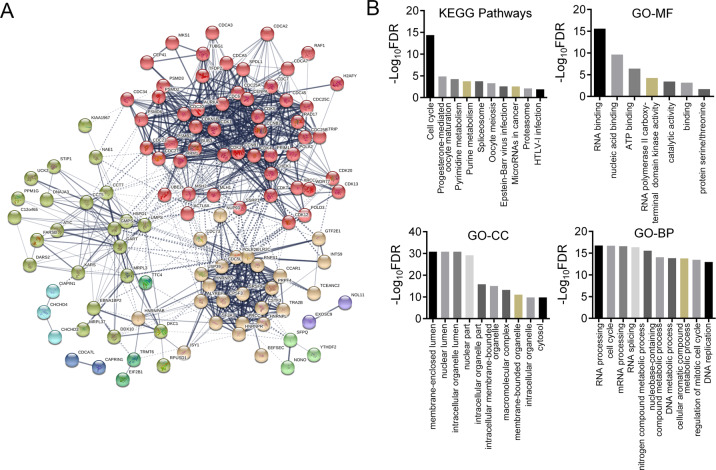
*Pontin* co-expressed gene network indicates the involvement of Pontin in cell cycle and RNA processing regulations. **A** Protein–protein interaction network of *Pontin* co-expressed genes in GBM screened by cBioportal and depicted by STRING. **B** KEGG pathway and GO enrichment (MF molecular function, CC cellular component, BP biological process) analyses of *Pontin* co-expressed genes by STRING. –Log_10_ transformed false discovery rates (FDR) are plotted for each enriched functional category.

To understand the role of Pontin in glioma, we screened out the *Pontin* co-expressed genes from the GBM gene expression profiling database in cBioPortal by setting the absolute value of Spearman’s correlation coefficient >0.5. A total of 19,942 genes were identified to be *Pontin* co-expressed genes. Among these genes, the top 237 genes were submitted to STRING to construct the protein–protein interaction network to identify the possible biological processes Pontin might act in (Fig. 2A). KEGG pathway analysis revealed that the *Pontin* co-expressed genes were functionally enriched in pathways like cell cycle, progesterone-mediated oocyte maturation, pyrimidine metabolism, spliceosome, and microRNAs in cancer (Fig. 2B). In addition, GO analysis for these co-expressed genes indicated their functional association with processes like regulation of the RNA processing, cell cycle, mRNA processing, RNA splicing, and nitrogen compound metabolic process (Fig. 2B). Notably, *Pontin* co-expressed genes were most closely associated with cell cycle control, suggesting that Pontin may affect glioma development through regulating the cell cycle. Besides, its relevancy with RNA processing or splicing was also noteworthy.

### Knockdown of Pontin inhibited cell growth and cell cycle progression in GBM cells

**Fig. 3:**
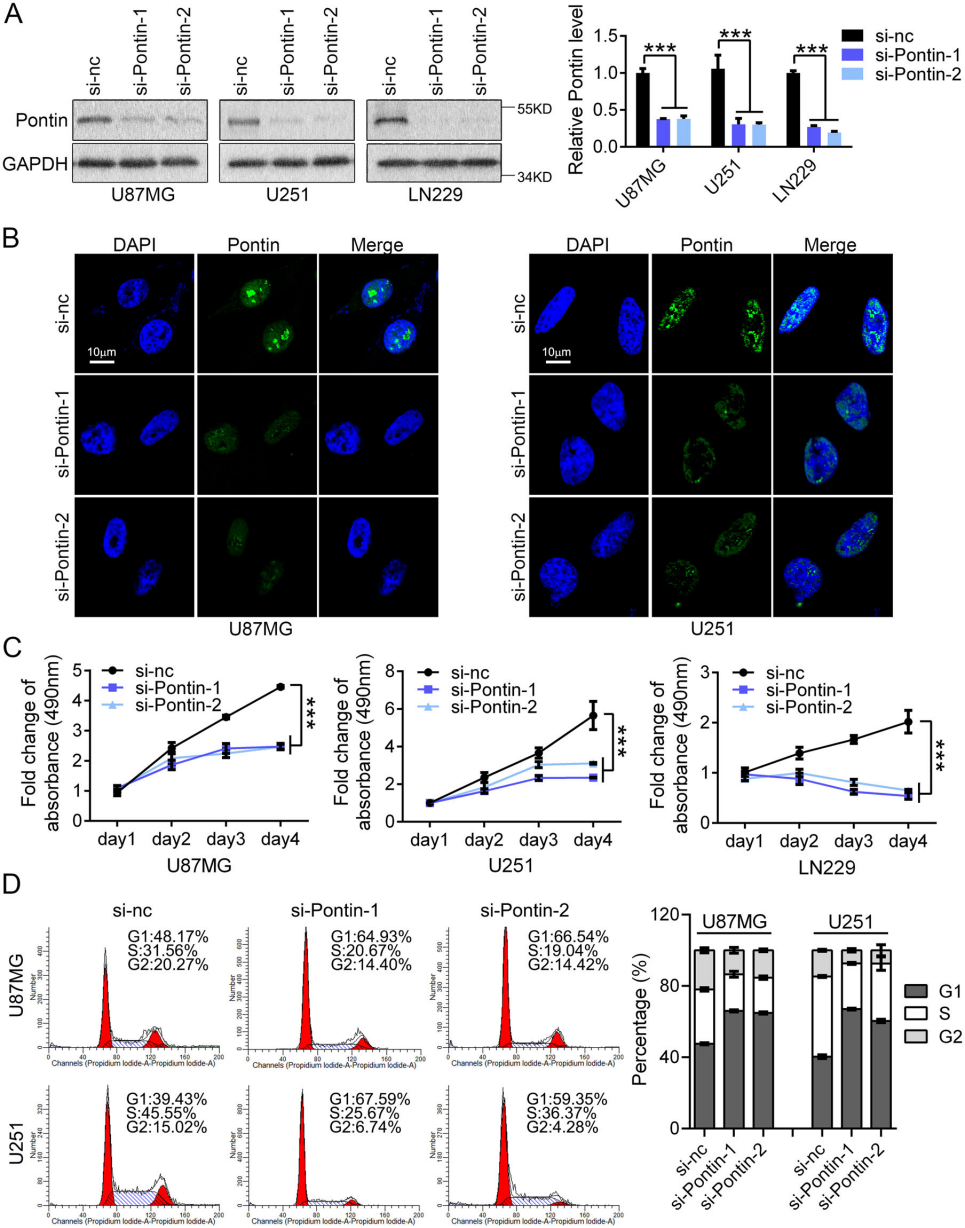
Knockdown of Pontin inhibits GBM cell growth by inducing cell cycle arresting. **A** Western blot of Pontin expression in U87MG, U251 cells, and LN229 transfected with indicated siRNAs (si-nc, si-Pontin-1, or si-Pontin-2). **B** IF detection of the expression and subcellular location of Pontin in U87MG and U251 as transfected in (**A**). Scale bar, 10 μm. **C** The growth curves of the indicated cells assessed by MTS assays. **D** FCM cell cycle analysis results of the indicated cells (left) and the percentages of cells in each phase (right). All the experiments were performed at least in triplicate. Data in (**A**), (**C**), and (**D**) are presented as the mean ± SD. ***P < 0.001.

To determine the biological roles of Pontin in gliomas, siRNAs were adopted to silence the endogenous Pontin in three GBM cell lines (U87MG, U251, LN229; Fig. 3A). IF analysis using the Pontin antibody revealed that Pontin localized in the nucleus, extremely assembling in the nucleolar dots in the control group (treated with negative control siRNA; si-NC), while Pontin siRNA transfection led to the obvious reduction of these IF signals (Fig. 3B). MTS assays were then performed to examine the effect of Pontin knockdown on cell growth, and the results showed that Pontin knockdown significantly inhibited cell growth in the three GBM cell lines tested (Fig. 3C). Moreover, flow cytometry analysis assessing the cellular DNA synthesis and cell cycle distribution revealed that Pontin knockdown increased the percentages of cells in the G1 peak, and decreased the percentages of the S or G2 peak (Fig. 3D and Supplementary Fig. S2). Collectively, these results indicated that Pontin contributed to GBM cell growth in vitro by facilitating cell cycle (G1) progression.

### Knockdown of Pontin inhibited the expression of E2F1 target genes

**Fig. 4:**
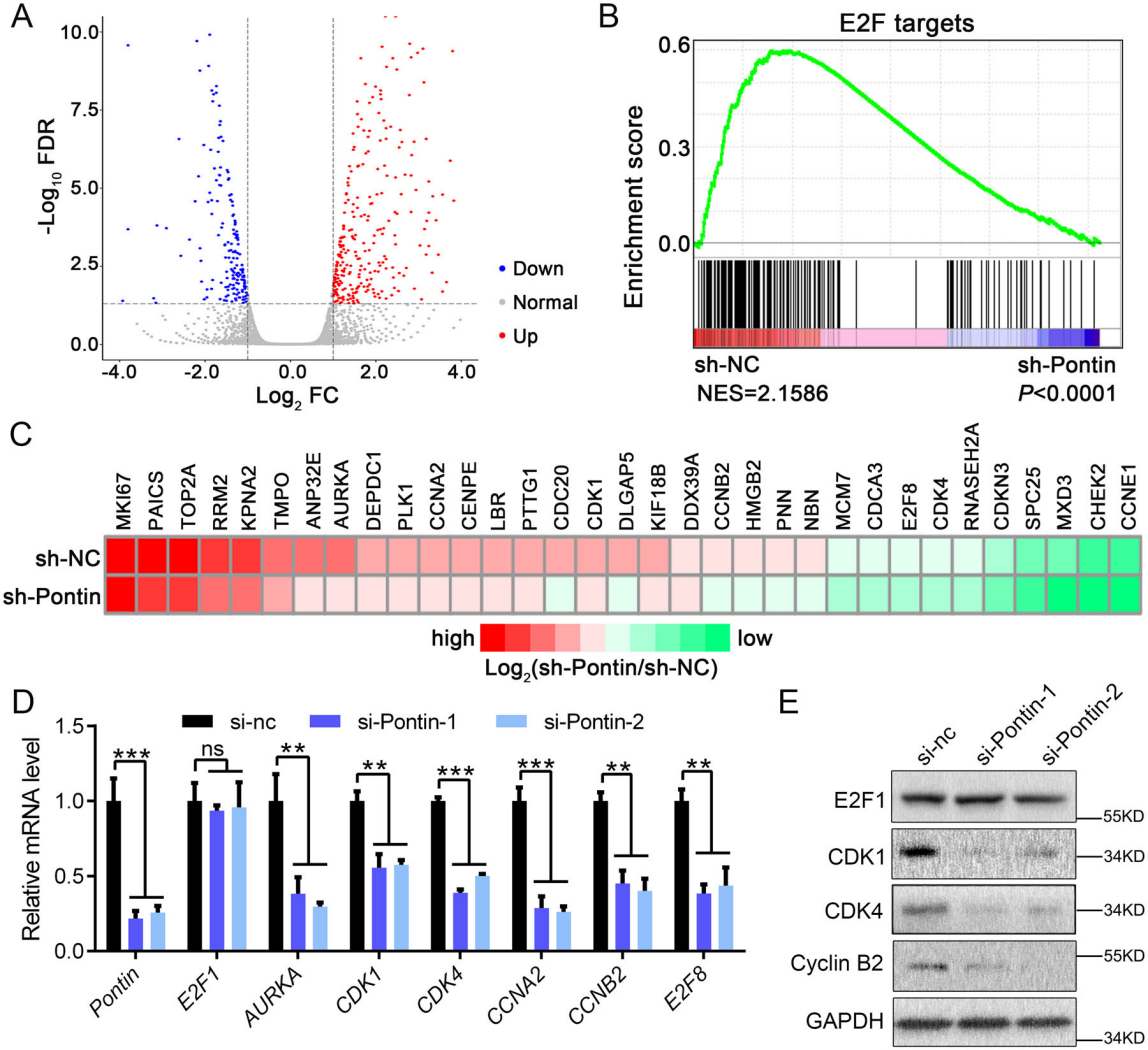
Pontin knockdown majorly affects the E2F1 transcription response. **A** Volcano plot for the differential expressed genes affected by Pontin knockdown screened by RNA-seq with FC > 2 and FDR < 0.05. **B** GSEA plot indicated a significant correlation between Pontin and E2F target expressions. **C** Heatmap of the top 33 E2F1 targets expressions in U87MG cells affected by Pontin knockdown. **D** qRT-PCR detection of *Pontin*, *E2F1*, *AURKA*, *CDK1*, *CDK4*, *CCNA2*, *CCNB2*, and *E2F8* expressions in U87MG cells as transfected in Fig. 3A. **E** Western blot analyses of E2F1, CDK1, CDK4, and Cyclin B2 expressions in U87MG cells as transfected in Fig. 3A. Experiments in (**D**) and (**E**) were performed at least in triplicate. Data in (**D**) are presented as the mean ± SD. ns *>*0.05; **P < 0.01; ***P < 0.001.

To further understand the global impact of Pontin knockdown on gene expression in glioma, we conducted high throughput sequencing of RNA (RNA-seq) on the control (sh-NC) and Pontin knockdown (sh-Pontin) groups of U87MG sub-cell lines. We observed a wide range of differential gene expression between the two groups (Fig. 4A). Gene set enrichment analysis (GSEA) indicated a significant correlation between Pontin and E2F target expressions (Fig. 4B, C). As is known, transcription factor E2F1 and its targets are crucial for G1/S transition^18,19^. qRT-PCR and Western blot confirmed that Pontin knockdown resulted in a marked downregulation of the expressions of *AURKA*, *CDK1*, *CDK4*, *CCNA2*, *CCNB2*, and *E2F8*, the six known targets of E2F1, while failed to regulate E2F1 itself expression (Figs. 4D, E). All these cell cycle-related E2F1 targets can promote cell passage through the G1/S checkpoint. These results demonstrated that Pontin knockdown induced cell cycle arrest at the G1 phase by inhibiting the expression of the E2F1 targets. Besides, the positive correlation between Pontin and six E2F1 targets (*AURKA*, *CDK1*, *CDK4*, *CCNA2*, *CCNB2*, *E2F8*) assessed by GEPIA in glioma further supported the positive regulation of E2F1 targets expressions by Pontin (Supplementary Fig. S3).

### Recruitment of pontin by E2F1 in GBM cells amplified the E2F1 transcription response

**Fig. 5:**
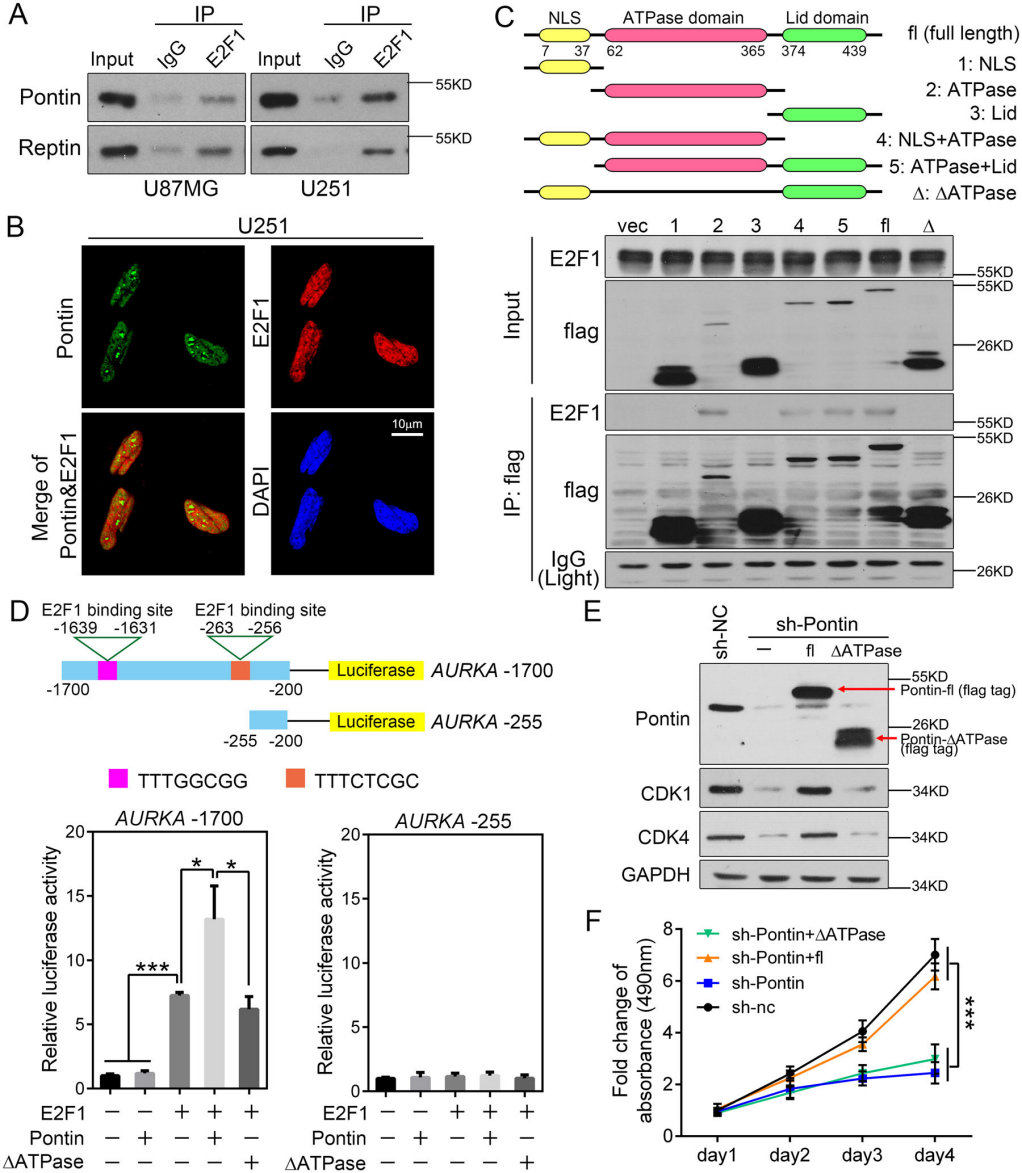
Pontin functions as a transcription co-activator of E2F1 in GBM cells. **A** Co-IP using IgG or E2F1 antibody in U87MG and U251 cell extracts. Endogenous Pontin and Reptin were pulled-down and detected in the pulled-down fractions by immunoblot. **B** Co-localization of endogenous Pontin (green) and E2F1 (red) in U251 cells. Representative images from biological triplicate experiments are shown. Scale bar, 10 μm. **C** Top: Schematic diagram of Pontin domains and construction of Pontin domain deleting mutants. All mutants are flag tagged. Bottom: Co-IP using the flag antibody in U87MG cell extracts. Interactions between flag tagged Pontin proteins and endogenous E2F1 were confirmed by immunoblot. **D** Luciferase assay was performed with reporters driven by *AURKA* promoter containing two E2F1-binding elements (*AURKA* −1700) or deleting E2F1-binding region (*AURKA* –255). Effect of Pontin or the Pontin ΔATPase (the ATPase domain deficient mutant) expression on *AURKA* –1700 or *AURKA* –255 promoter-luciferase activity in the presence or absence of E2F1 was shown. Luciferase activities were normalized by β-galactosidase activity. Values are expressed as mean ± SD of three independent experiments. *P < 0.05; ***P < 0.001. **E** Western blot analyses of Pontin, CDK1, and CDK4 expressions in stable U87MG sub-cell lines (sh-NC, sh-Pontin) transfected with indicated plasmids. **F** The growth curves of the indicated cells assessed by MTS assays. Data are presented as the mean ± SD. ***P < 0.001.

As Pontin/Reptin may function as co-activators for many transcription factors, including E2F1^20,21^, we then checked the possibility whether Pontin/Reptin could bind to E2F1 and form a specific regulatory module for the downstream targets in GBM cells. Co-IP revealed a mutual binding of endogenous Pontin/Reptin and E2F1 in U87MG and U251 cells (Fig. 5A). IF detection also indicated that endogenous Pontin and E2F1 co-localized in the nucleus, but the majority outside of the nucleolar dots, where Pontin assembled (Fig. 5B), suggesting distinct roles of Pontin in disparate locations. Specifically, we found that the binding of Pontin with E2F1 depended on the presence of its ATPase domain since only the flag tagged Pontin mutants containing the ATPase domain precipitated with the endogenous E2F1, while the ATPase domain deficient mutant failed (Fig. 5C). Next, we performed luciferase reporter assays with reporters derived from *AURKA* promoter containing (*AURKA* −1700) or deleting (*AURKA* −255) E2F1 binding sites (Fig. 5C). Overexpression of E2F1 observably increased *AURKA* −1700 promoter luciferase activity, and introduction of the wild-type Pontin reinforced this increase by nearly twofold, but introducing the ATPase domain deficient mutant of Pontin failed to produce the augmentation (Fig. 5D). Notably, the introduction of Pontin alone failed to activate the *AURKA* −1700 reporter (Fig. 5D). The *AURKA* −255 reporter without E2F1 binding sites did not respond to the overexpression of either E2F1 or Pontin (Fig. 5D). Furthermore, restoration of the shRNA-resistant full-length Pontin (sh-Pontin + fl) efficiently reversed the adverse effects of Pontin knockdown on E2F1 targets (CDK1, CDK4) expressions and cell growth. However, the ATPase domain deficient mutant of Pontin failed to exert any “rescue” effects (Fig. 5E, F). Collectively, these results implied that recruitment of Pontin by E2F1 in GBM cells amplified the E2F1 transcription response, in other words, Pontin acted as a co-activator for E2F1. The ATPase activity majorly contributed to the E2F1 interaction and the thereafter transcription amplification and growth promotion.

### The Pontin-affected alternative splicing landscape in GBM cells

**Fig. 6:**
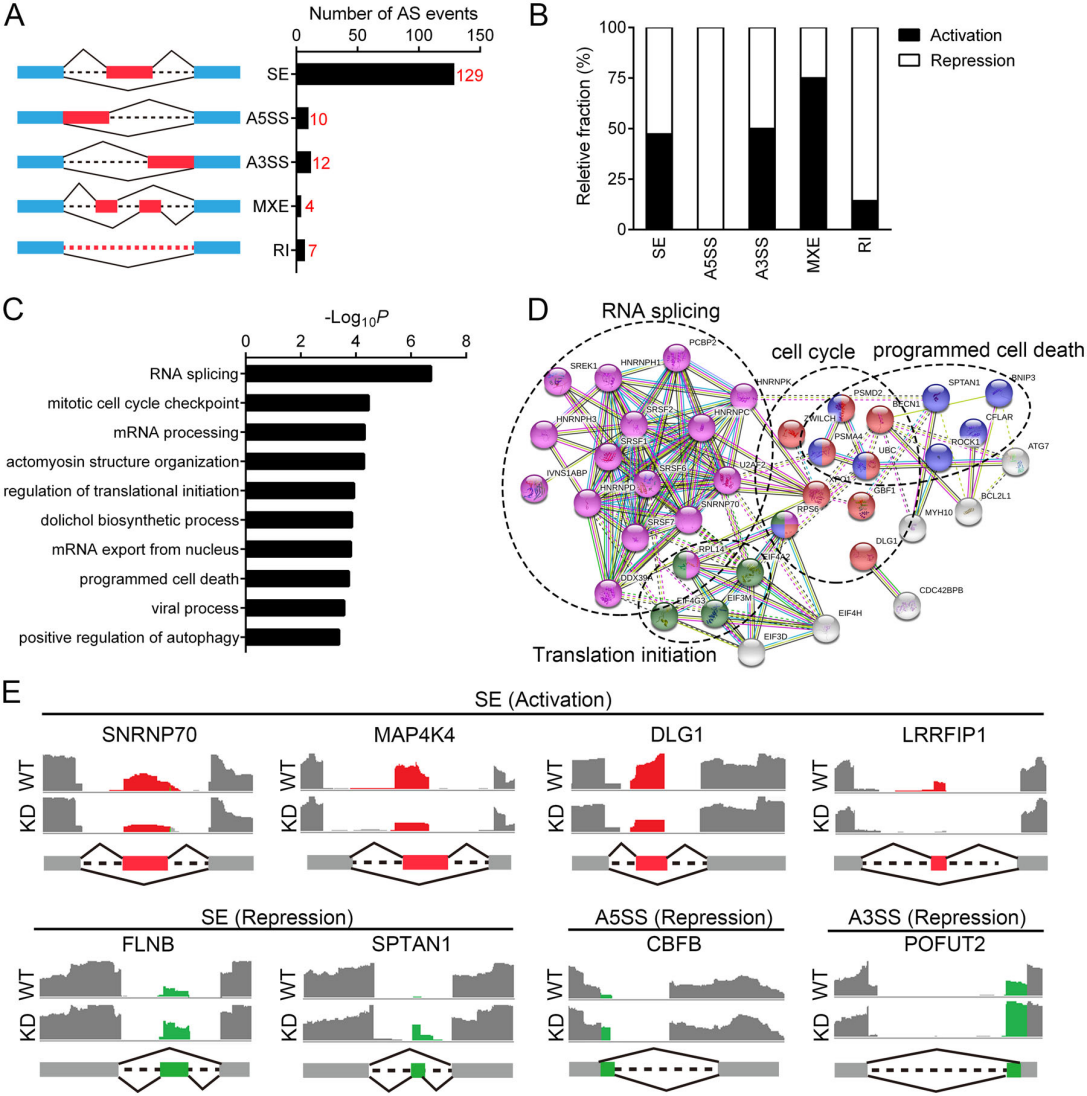
Global profiles of Pontin-affected AS in U87MG cells. **A** Diagrams of the exclusion and inclusion isoforms for the five modes of AS events examined (left) and the graph of Pontin-affected AS events in each category (right). SE skipped exon, A5SS alternative 5’ splice site, A3SS alternative 3’ splice site, MXE mutually exclusive exon, RI retained intron. **B** Relative fraction of AS events affected positively (activation) or negatively (repression) by Pontin in each category. **C** GO annotation results of the AS targets affected by Pontin knockdown. –Log_2_ transformed fisher exact *P* values are plotted for each enriched functional category. **D** Functional association network of the Pontin-affected AS targets. Genes incorporated in (**C**) were analyzed using the STRING database, and the subgroups are marked according to their functions. **E** Pontin can promote both exon inclusion and exclusion in vivo. Pontin-activated cassette exons (upper) and Pontin-repressed cassette exons (bottom) identified by RNA-seq are schematically diagrammed with mapped RNA-seq reads that cover the corresponding exons. Colored boxes, alternative exons; dashed lines, introns; gray boxes, flanking constitutive exons.

In retrospect, the *Pontin* co-expressed gene network analysis also suggested that Pontin might be a novel splicing regulator in glioma, since GO analysis for these co-expressed genes indicated their functional enrichment in processes like regulation of the RNA processing, mRNA processing, and RNA splicing (Fig. 2B). To understand the impact of Pontin on global AS in GBM cells, we screened out its downstream AS events by analyzing the RNA-seq data obtained in the control (sh-NC) and Pontin knockdown (sh-Pontin) groups of U87MG sub-cell lines as described above. Data mining showed that the majority of these AS events belonged to the skipped exon (SE) category (Fig. 6A). Subsequent analysis indicated the dual role of Pontin as a splicing activator and repressor, as it induced both exon/intron inclusion (activation) and exclusion (repression; Fig. 6B). Moreover, these Pontin-affected AS targets were functionally associated with cancer-related functions, such as RNA splicing, mitotic cell cycle checkpoint, programmed cell death, and so on (Fig. 6C, D). Representative examples of eight AS events were shown in Fig. 6E, indicating that Pontin displayed both splicing activation (*SNRNP70, MAP4K4, DLG1*, and *LRRFIP1*) and repression (*FLNB, SPTAN1, CBFB,* and *POFUT2*) effects on distinct targets belonging to different AS categories. These results proposed that the oncogenic roles of Pontin are also associated with its modulating effects on AS.

## Discussion

Our present work represents a preliminary study of the AAA + ATPase Pontin and its downstream gene regulation landscape in glioma. During this study, we identified that Pontin was overexpressed in gliomas and possessed tumor-promoting capacities as a transcription co-activator of E2F1, thereby controlling cell cycle progression and cell growth. The involvement of Pontin in AS regulation might also contribute to its oncogenic role in glioma. Therefore, we concluded that Pontin promoted gliomagenesis at least by amplifying the E2F1 transcription response.

A large body of evidence has proved the overexpression of Pontin in human cancers, including in hepatocellular carcinoma, colorectal cancer, breast cancer, and lung cancer^8,16^. However, its expression, clinical significance, and biological roles in gliomas remain largely unknown. Herein, our first data mining on GEPIA and Oncomine revealed an overexpression of Pontin in gliomas. Importantly, we found that higher Pontin expression correlated with the worse outcome of the glioma patients, implying the potential application of Pontin as a novel prognostic factor for glioma. Detection of Pontin mRNA and protein expression in gliomas further confirmed its overexpression.

As is known, Pontin is involved in various cellular processes that are important for oncogenesis including transcription regulation, chromosome remodeling, DNA damage repair, assembly of macromolecular complexes, regulation of cell cycle activity, and cell movement, etc^8^. All these complex cellular regulatory functions of Pontin ultimately lead to the promotion of cancer cell proliferation and survival^12^. Through *Pontin* co-expressed gene network analysis, we recognized that Pontin was associated with various cancer-related biological functions in glioma, including the RNA processing, cell cycle, and other signaling pathways. Consistently, our in vitro studies verified that knockdown of Pontin inhibited glioma cell growth and cell cycle progression. Specifically, endogenous Pontin inhibition led to cell cycle arrest at the G1/S phase checkpoint, thus resulting in the accumulation of cells in G1 and a reduction of cells in all other phases of the cell cycle. Therefore, we considered that Pontin promoted gliomagenesis and tumor progression at least by facilitating cell cycle processing and cell growth, although our extensive in vitro and in vivo work is still on the way to confirm the oncogenic roles of Pontin in glioma.

Using RNA-seq, we found that Pontin knockdown mainly affected the E2F target expression. The following qRT-PCR and Western blot confirmed the positive regulation of E2F1 target expression by Pontin. As Pontin and its close family member, Reptin is known to physically interact with multiple transcription factors with a strong involvement in carcinogenesis, and function as their co-activators to increase the corresponding transcription response^8,16^, we then tested whether Pontin cooperated with E2F1 to activate the transcription of the downstream targets of E2F1 in glioma. The results came out that Pontin interacted with E2F1, and this interaction depended on the presence of the ATPase domain of Pontin. Results from the luciferase reporter assay and the rescue experiments demonstrated that Pontin increased E2F1-dependent transcription and promoted GBM cell growth in an ATPase domain-dependent manner.

Currently, there is a debate whether human Pontin alone has an intrinsic ATPase activity^31–33^. Multiple lines of evidence supporting the ATPase activity of Pontin come from experiments where critical amino acids within Walker A or Walker B motif (within the ATPase domain) were mutated. These mutations could abrogate the functional properties of Pontin in promoting cell growth, and abolish the expression elevation of phosphatidylinositol 3-kinase-related protein kinases (PIKKs)^34–36^. Pontin was also found to be essential for MYC-mediated oncogenic transformation and modulated MYC-induced apoptosis in an ATPase-dependent manner, where the ATPase-deficient mutant of Pontin enhanced apoptosis if MYC was overexpressed^15^. In addition, the ATPase activities are also required for clonogenesis and survival of the cancers of white blood cells^37,38^. Our results suggested that Pontin bound to E2F1 relying on the presence of the ATPase domain, which contributed majorly for the following transcription response amplification and growth promotion of Pontin in glioma. These findings were fully in line with the previous data characterizing the importance of the ATPase activity of Pontin. Developing Pontin as the target in glioma would be especially interesting since it might be a “druggable” target because of the ATPase activity.

AS of pre-mRNAs plays a pivotal role for the establishment and maintenance of distinct human cell types^39^. Conversely, dysregulation of AS occurs in many genetic diseases and cancers, and is sufficient to drive disease initiation, progression, and therapeutic response. During the past few decades, substantial effort has been devoted to identify and characterize the splicing factors that control AS programs in both healthy and diseased cells. For the first time, we found that Pontin was implicated in AS regulation and could act as either a splicing activator or repressor to control numerous cancer-related AS targets in glioma. This was not striking since previous reports have established that Pontin was involved in snRNP assembly or trafficking^25^, and its family member Reptin could also modulate AS program^26^. Further studies are underway to understand the detailed mechanisms of Pontin in controlling AS in glioma, which requires extensive efforts in the future.

Taken together, our investigation on Pontin uncovered its oncogenic roles and complex mechanisms in promoting glioma cell growth. We anticipate that cooperation with E2F1 to increase the transcription response stands for an important but not the only mechanism interpreting the tumor-promoting capacities of Pontin. More research is still required, aiming at convincingly demonstrating that Pontin is an indeed active ATPase, and that the ATPase activity is important for its oncogenic functions. Nevertheless, our findings demonstrate that Pontin is a vital cell cycle regulator at least by amplifying E2F1 transcription response in glioma, shedding light on the possibility of targeting Pontin for anti-glioma therapy.

## Supplementary information

Supplemental Material

## Data Availability

All data are available upon request of the corresponding author.

## References

[1] Brodbelt A (2015). Glioblastoma in England: 2007-2011. Eur. J. Cancer.

[2] Omuro A, DeAngelis LM (2013). Glioblastoma and other malignant gliomas: a clinical review. JAMA.

[3] Louis DN (2016). The 2016 World Health Organization Classification of Tumors of the Central Nervous System: a summary. Acta Neuropathol..

[4] Wen PY, Kesari S (2008). Malignant gliomas in adults. N. Engl. J. Med..

[5] Lukas RV, Boire A, Nicholas MK (2007). Emerging therapies for malignant glioma. Expert. Rev. Anticancer Ther..

[6] Lin L, Cai J, Jiang C (2017). Recent Advances in Targeted Therapy for Glioma. Curr. Med. Chem..

[7] Matias PM (2015). The AAA+ proteins Pontin and Reptin enter adult age: from understanding their basic biology to the identification of selective inhibitors. Front. Mol. Biosci..

[8] Mao YQ, Houry WA (2017). The Role of Pontin and Reptin in Cellular Physiology and Cancer Etiology. Front. Mol. Biosci..

[9] Kim JH (2005). Transcriptional regulation of a metastasis suppressor gene by Tip60 and beta-catenin complexes. Nature.

[10] Bauer A, Huber O, Kemler R (1998). Pontin52, an interaction partner of beta-catenin, binds to the TATA box binding protein. Proc. Natl Acad. Sci. USA.

[11] Bellosta P (2005). Myc interacts genetically with Tip48/Reptin and Tip49/Pontin to control growth and proliferation during Drosophila development. Proc. Natl Acad. Sci. USA.

[12] Huber O (2008). Pontin and reptin, two related ATPases with multiple roles in cancer. Cancer Res..

[13] Jha S, Gupta A, Dar A, Dutta A (2013). RVBs are required for assembling a functional TIP60 complex. Mol. Cell. Biol..

[14] Feng Y, Lee N, Fearon ER (2003). TIP49 regulates beta-catenin-mediated neoplastic transformation and T-cell factor target gene induction via effects on chromatin remodeling. Cancer Res..

[15] Wood MA, McMahon SB, Cole MD (2000). An ATPase/helicase complex is an essential cofactor for oncogenic transformation by c-Myc. Mol. Cell.

[16] Grigoletto A, Lestienne P, Rosenbaum J (2011). The multifaceted proteins Reptin and Pontin as major players in cancer. Biochim. Biophys. Acta.

[17] Otto T, Sicinski P (2017). Cell cycle proteins as promising targets in cancer therapy. Nat. Rev. Cancer.

[18] Johnson J (2016). Targeting the RB-E2F pathway in breast cancer. Oncogene.

[19] Malumbres M, Barbacid M (2001). To cycle or not to cycle: a critical decision in cancer. Nat. Rev. Cancer.

[20] Taubert S (2004). E2F-dependent histone acetylation and recruitment of the Tip60 acetyltransferase complex to chromatin in late G1. Mol. Cell. Biol..

[21] Tarangelo A (2015). Recruitment of Pontin/Reptin by E2f1 amplifies E2f transcriptional response during cancer progression. Nat. Commun..

[22] Baralle FE, Giudice J (2017). Alternative splicing as a regulator of development and tissue identity. Nat. Rev. Mol. Cell Biol..

[23] Oltean S, Bates DO (2014). Hallmarks of alternative splicing in cancer. Oncogene.

[24] Chen J, Weiss WA (2015). Alternative splicing in cancer: implications for biology and therapy. Oncogene.

[25] Kakihara Y, Saeki M (2014). The R2TP chaperone complex: its involvement in snoRNP assembly and tumorigenesis. Biomol. Concepts.

[26] Cloutier P (2017). R2TP/Prefoldin-like component RUVBL1/RUVBL2 directly interacts with ZNHIT2 to regulate assembly of U5 small nuclear ribonucleoprotein. Nat. Commun..

[27] Zhou X (2017). Quaking-5 suppresses aggressiveness of lung cancer cells through inhibiting beta-catenin signaling pathway. Oncotarget.

[28] Zhou X (2019). Splicing factor SRSF1 promotes gliomagenesis via oncogenic splice-switching of MYO1B. J. Clin. Invest..

[29] Luo W (2019). miR-135a-5p Functions as a Glioma Proliferation Suppressor by Targeting Tumor Necrosis Factor Receptor-Associated Factor 5 and Predicts Patients’ Prognosis. Am. J. Pathol..

[30] Zhou X (2014). Transcriptome analysis of alternative splicing events regulated by SRSF10 reveals position-dependent splicing modulation. Nucleic Acids Res..

[31] Matias PM, Gorynia S, Donner P, Carrondo MA (2006). Crystal structure of the human AAA+ protein RuvBL1. J. Biol. Chem..

[32] Qiu XB (1998). An eukaryotic RuvB-like protein (RUVBL1) essential for growth. J. Biol. Chem..

[33] Choi J, Heo K, An W (2009). Cooperative action of TIP48 and TIP49 in H2A.Z exchange catalyzed by acetylation of nucleosomal H2A. Nucleic Acids Res..

[34] Jonsson ZO (2001). Rvb1p and Rvb2p are essential components of a chromatin remodeling complex that regulates transcription of over 5% of yeast genes. J. Biol. Chem..

[35] King TH, Decatur WA, Bertrand E, Maxwell ES, Fournier MJ (2001). A wellconnected and conserved nucleoplasmic helicase is required for production of box C/D and H/ACA snoRNAs and localization of snoRNP proteins. Mol. Cell. Biol..

[36] Izumi N (2010). AAA+ proteins RUVBL1 and RUVBL2 coordinate PIKK activity and function in nonsense-mediated mRNA decay. Sci. Signal..

[37] Osaki H (2013). The AAA+ ATPase RUVBL2 is a critical mediator of MLL-AF9 oncogenesis. Leukemia.

[38] Breig O (2014). Pontin is a critical regulator for AML1-ETO-induced leukemia. Leukemia.

[39] Dvinge H, Guenthoer J, Porter PL, Bradley RK (2019). RNA components of the spliceosome regulate tissue- and cancer-specific alternative splicing. Genome Res..

