# Supplementary material for: The ATPase Pontin is a key cell cycle regulator by amplifying E2F1 transcription response in glioma
Source: Cell Death Dis. 2021 Feb 1;12(2):141. doi: 10.1038/s41419-021-03421-4 (PMC7862657; doi:10.1038/s41419-021-03421-4)
Supplement: Supplementary file 1 — Supplemental Material [file 41419_2021_3421_MOESM1_ESM.pdf]

Supplementary Figures and Tables

Supplementary Figure S1

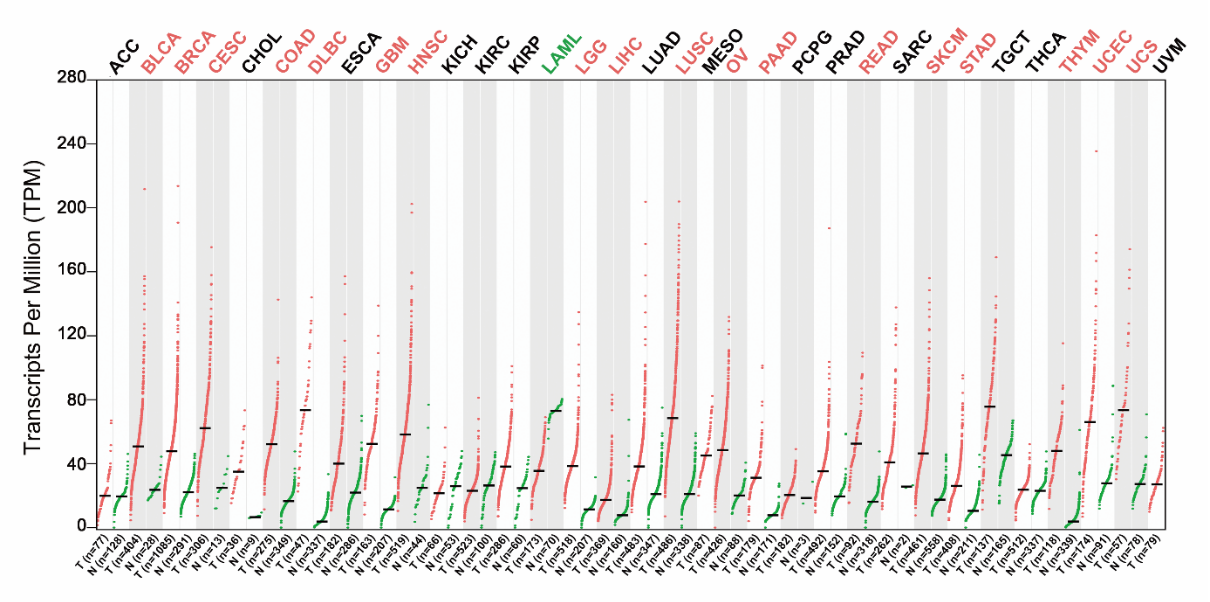

Supplementary Figure S1. Data from GEPIA shows that *Pontin* is overexpressed in various cancer types. Each dots represent expression of samples. Abbreviations of the cancers are given in the X axial (upper), in which red fonts stand for uregulation, green fonts downregulation, and black fonts constant. Numbers of tumors (T, in red) and normal controls (N, in green) are given in brackets of the X axial (bottom). Short transverse lines represent the mean values of each group. The full names of the cancers are: ACC, Adrenocortical carcinoma; BLCA, Bladder Urothelial Carcinoma; BRCA, Breast invasive carcinoma; CESC, Cervical squamous cell carcinoma and endocervical adenocarcinoma; CHOL, Cholangio carcinoma; COAD, Colon adenocarcinoma; DLBC, Lymphoid Neoplasm Diffuse Large B-cell Lymphoma; ESCA, Esophageal carcinoma; GBM, Glioblastoma multiforme; HNSC, Head and Neck squamous cell carcinoma; KICH, Kidney Chromophobe; KIRC, Kidney renal clear cell carcinoma; KIRP, Kidney renal papillary cell carcinoma; LAML, Acute Myeloid Leukemia; LGG, Brain Lower Grade Glioma; LIHC, Liver hepatocellular carcinoma; LUAD, Lung adenocarcinoma; LUSC, Lung squamous cell

carcinoma; MESO, Mesothelioma; OV, Ovarian serous cystadenocarcinoma; PAAD, Pancreatic adenocarcinoma; PCPG, Pheochromocytoma and Paraganglioma; PRAD, Prostate adenocarcinoma; READ, Rectum adenocarcinoma; SARC, Sarcoma; SKCM, Skin Cutaneous Melanoma; STAD, Stomach adenocarcinoma; TGCT, Testicular Germ Cell Tumors; THCA, Thyroid carcinoma; THYM, Thymoma; UCEC, Uterine Corpus Endometrial Carcinoma; UCS, Uterine Carcinosarcoma; UVM, Uveal Melanoma.

Supplementary Figure S2

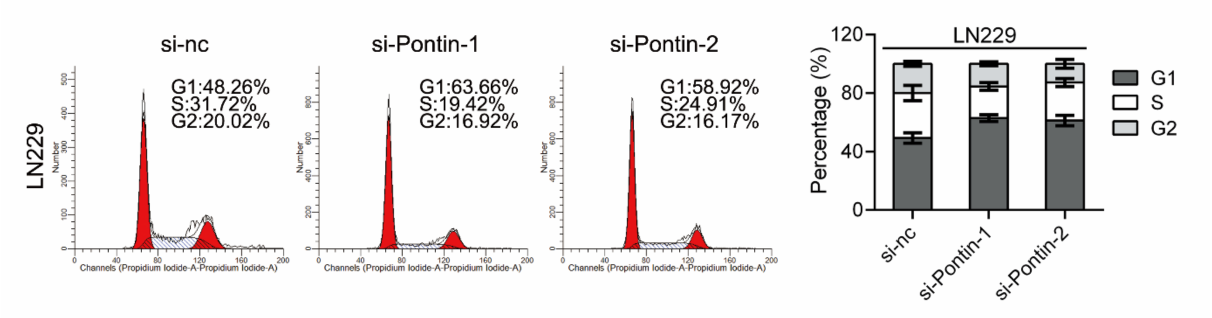

Supplementary Figure S2. Knockdown of Pontin induces cell cycle retardation in LN229 cells. FCM cell cycle analysis results of the LN229 cells transfected with indicated siRNA (left) and the percentages of cells in each phase (right). The experiments were repeated three times. Data are presented as the mean ± SD.

Supplementary Figure S3

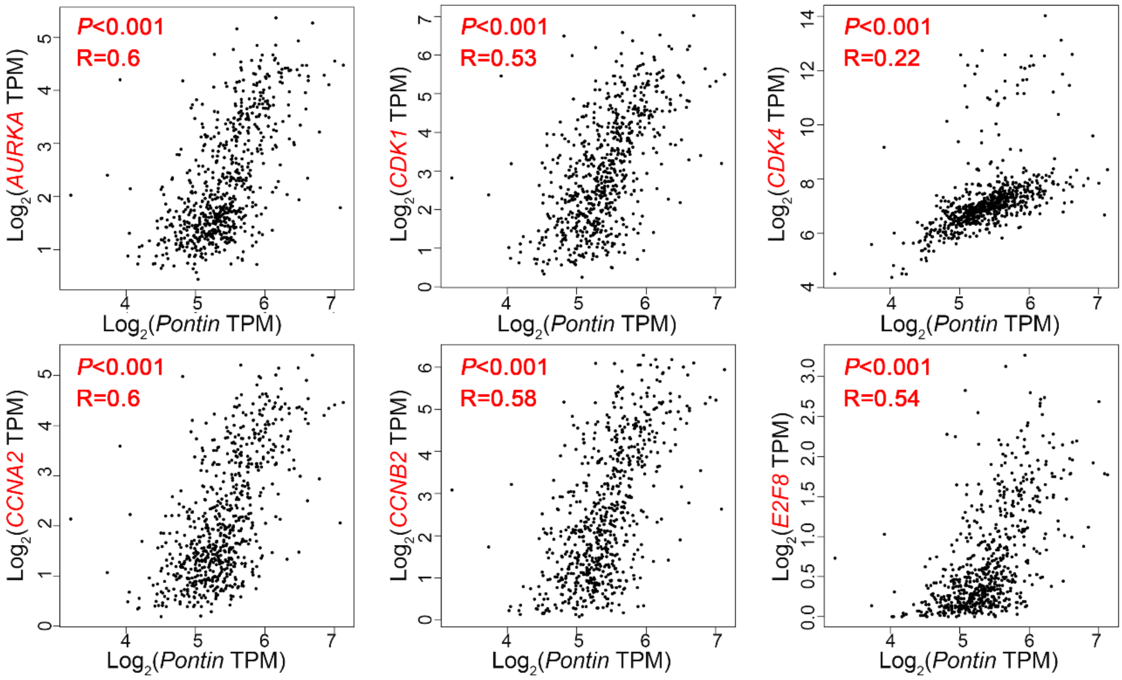

Supplementary Figure S3. The correlation between *NEAT1* and *E2F1*, *E2F2*, *MYBL2*, *CCND1*, *CDK2* and *CDK6* mRNA expression. Correlation analysis is accomplished using 163 GBM tumors and 518 LGG tumors collectively in GEPIA dataset (http://gepia.cancer-pku.cn). *P*<0.001 by Spearman correlation test.

Supplementary Table S1. Sequences of qRT-PCR primers

Name	Sequences (5'-3')
**Pontin-Forward**	CGGGTGTGCTGTTTGTTGAT
**Pontin-Reverse**	AAGATGACGATGGGAGCGATA
**ACTB-Forward**	GATCATTGCTCCTCCTGAGC
**ACTB-Reverse**	ACTCCTGCTTGCTGATCCAC
**AURKA-Forward**	GCCATCGGCACCTGAAAATA
**AURKA-Reverse**	CCCACTGCCTCTTTTTTGATTC
**E2F1-Forward**	CCGCCATCCAGGAAAAGG
**E2F1-Reverse**	AGCGCTTGGTGGTCAGATTC
**CCNB2-Forward**	GCATGCGTGCCATCCTAGT
**CCNB2-Reverse**	CTGGCTGAACCTGTAAAAATCGA
**CCNA2-Forward**	CCTGCGTTCACCATTCATGT
**CCNA2-Reverse**	CAGGGCATCTTCACGCTCTAT
**E2F8-Forward**	GGCCCAGAAATCAGTCCAAAT
**E2F8-Reverse**	GACCGTCTCACCTCCAAATCA
**CDK1-Forward**	CCAATAATGAAGTGTGGCCAGAA
**CDK1-Reverse**	ATGCTAGGCTTCCTGGTTTCC
**CDK4-Forward**	GGCGACTGGAGGCTTTTGA
**CDK4-Reverse**	TCAAACACCAGGGTTACCTTGA

Supplementary Table S2. Sequences of cloning primers

Name	Sequences (5'-3')
**Pontin-NLS-BamH1-F**	CGCGGATCCATGAAGATTGAGGAGGTGAAGAG
**Pontin-NLS-Hind3-R**	CCCAAGCTTCATTTTCTTGCTTTTGAT
**Pontin-ATPase-BamH1-F**	CGCGGATCCATGGCTGGAAGAGCTGTCTTG
**Pontin-ATPase-Hind3-R**	CCCAAGCTTCTGTTTCATTTCCTGTGGAGT
**Pontin-lid-BamH1-F**	CGCGGATCCATGCCACAGGAAATGAAACAG
**Pontin-lid-Hind3-R**	CCCAAGCTTTCACTTCATGTACTTATCCTGC
**Pontin-ΔATPase-F**	CAAAAGCAAGAAAATGTATACTCCACAGGAAATG
**Pontin-ΔATPase-R**	CATTTCCTGTGGAGTATACATTTTCTTGCTTTTG
**Pontin-3.1-Hind3-Forward**	CCCAAGCTTTATGAAGATTGAGGAGGTGAAG
**Pontin-3.1-BamH1-Reverse**	CGCGGATCCTCACACTTCATGTACTTATCCTGC
**E2F1-3.1- Hind3-Forward**	CCCAAGCTTTATGGCCTTGGCCGGGGCCCCT
**E2F1-3.1-BamH1-Reverse**	CGCGGATCCTCAGAAATCCAGGGGGGTGAG
**AURKA-1700-XhoI-F**	CCGCTCGAGTCCAGATCCCTGAGCTTAACC
**AURKA-255-XhoI-F**	CCGCTCGAGCATCTTACTTACTGGCACGTT
**AURKA-Hind3-R**	CCCAAGCTTTGGAGAGGAACCTGAAATAGAGG
